# N-benzyl-N-methyldecan-1-amine, derived from garlic, and its derivative alleviate 2,4-dinitrochlorobenzene-induced atopic dermatitis-like skin lesions in mice

**DOI:** 10.1038/s41598-024-56496-2

**Published:** 2024-03-21

**Authors:** Ji Eun Kim, Phatcharaporn Budluang, Jumin Park, Kon Ho Lee, Sirichatnach Pakdeepromma, Chutima Kaewpiboon, Ho Young Kang, Dae Youn Hwang, Young-Hwa Chung

**Affiliations:** 1 Department of Biomaterials Science (BK21 FOUR Program), College of Natural Resources and Life Science, Pusan National University, Miryang, 50463 Republic of Korea; 2 Department of Cogno-Mechatronics Engineering, Optomechatronics Research Institute, Pusan National University, Busan, Republic of Korea; 3 Department of Food Science and Nutrition, Pusan National University, Busan, 46241 Republic of Korea; 4 Department of Convergence Medical Science, Gyeongsang National University College of Medicine, Jinju, 52828 Republic of Korea; 5 Department of General Science and Liberal Arts, King Mongkut’s Institute of Technology Ladkrabang Prince of Chumphon Campus, Pathio, Chumphon, 86160 Thailand; 6 Department of Biology, Faculty of Science and Digital Innovation, Thaksin University, Phatthalung Campus, Phatthalung, 93210 Thailand; 7 Department of Microbiology, Pusan National University, Busan, 46241 Republic of Korea

**Keywords:** N-benzyl-N-methyldecan-1-amine, 2,4-Dinitrochlorobenzene, Atopic dermatitis, Inflammation, Mast cells, IgE titer, Pharmaceutics, Pharmacology, Drug discovery, Molecular medicine

## Abstract

Given the intricate etiology and pathogenesis of atopic dermatitis (AD), the complete cure of AD remains challenging. This study aimed to investigate if topically applying N-benzyl-N-methyldecan-1-amine (BMDA), derived from garlic, and its derivative [decyl-(4-methoxy-benzyl)-methyl-1-amine] (DMMA) could effectively alleviate AD-like skin lesions in 2,4-dinitrochlorobenzene (DNCB)-treated mice. Administering these compounds to the irritated skin of DNCB-treated mice significantly reduced swelling, rash, and excoriation severity, alongside a corresponding decrease in inflamed epidermis and dermis. Moreover, they inhibited spleen and lymph node enlargement and showed fewer infiltrated mast cells in the epidermis and dermis through toluidine-blue staining. Additionally, they led to a lower IgE titer in mouse sera as determined by ELISA, compared to vehicle treatment. Analyzing skin tissue from the mice revealed decreased transcript levels of inflammatory cytokines (TNF-α, IL-1β, and IL-6), IL-4, iNOS, and COX-2, compared to control mice. Simultaneously, the compounds impeded the activation of inflammation-related signaling molecules such as JNK, p38 MAPK, and NF-κB in the mouse skin. In summary, these findings suggest that BMDA and DMMA hold the potential to be developed as a novel treatment for healing inflammatory AD.

## Introduction

Atopic dermatitis (AD) is a skin disorder characterized by skin inflammation, disruption of the skin barrier, chronic itching, resulting in the development of rash or hives^1^. AD shows chronic relapses with intense itching, which worsens the quality of life for afflicted patients^2^. The etiology of AD has been proposed to involve several factors, including heightened immune sensitivity^3^, functional loss due to filaggrin gene mutation^4^, and microbial infections^5^. Mast cells play a crucial role in the progression of AD, comprising both acute and chronic phases^6^. B cells expressing IgE repeatedly expose allergens, leading to a drastic release of IgE. Since mast cells have high-density and high-affinity Fcε receptors on their cell surface, they secrete granules containing histamine, inflammatory cytokines such as IL-1β, IL-6, and prostaglandin E2 (PDE2) upon binding to IgE through Fcε receptors^7,8^. Mast cells are linked to Th2 cells, which dominate the chronic phase of AD^6^.

To induce allergic contact dermatitis, small-molecule haptens capable of easy skin penetration are introduced and repeatedly applied to the mouse’s skin in many studies^9–11^. This process enhances contact hypersensitivity responses, ultimately leading to the development of inflammation^12^. Despite the distinction between AD and hapten-induced dermatitis as separate entities, they exhibit some shared features including pruritus, disruption of the skin barrier, and inflammation triggered by activation of innate immunity^12–14^. Thus, DNCB-induced dermatitis model is adopted to mimic AD, terming it AD-like lesion, supported by various lines of evidence from other studies^15–17^.

All components isolated from garlic (*Allium sativum*) has been reported to possess “sulfur” atom in their molecular structure. These compounds include allicin, diallyl trisulfide, diallyl disulfide, diallyl sulfide, methyl allyl disulfide, and ajoene^18–20^. For example, a functional study reports that diallyl disulfide, a lipid-soluble organosulfur compound, inhibits the expression of COX2, iNOS2, and MMP-9, thereby reducing the production of inflammatory cytokines such as IL-1β and IL-6 with a murine macrophage RAW264.7 cell line^21^. These properties ultimately contribute to relieving ovalbumin-induced asthma in the mouse model^21^. Diallyl trisulfide also inhibits LPS-induced inflammation through the NF-κB pathway with an immortalized murine microglia cells^22^ and exhibits a preventive effect on oxidative and inflammatory damage in naphthalene-treated mice^23^. Recently, BMDA, a novel compound without sulfur atom originated from garlic cloves has been prepared using an organic solvent extraction method. It exhibits its ability to arrest the cell cycle due to decreased expression of cyclin-dependent kinase 2 and Cdc2^24^. Additionally, our study documents that BMDA, synthesized by a reductive amination method, restrains cancer stem cell-like phenotypes through down-regulation of both Akt-ERK1/2 and β-catenin signaling pathways in CUG2-overexpressing cells^25^.

This study aimed to explore whether BMDA and its derivative DMMA could alleviate DNCB-induced AD-like skin lesions. We initially demonstrate that topical application of BMDA or DMMA on the skin of DNCB-treated mice hinders the inflammatory progression of the disease, blocking the infiltration of mast cells and reducing IgE production. These outcomes may be attributed to decreased inflammatory mediators such as TNF-α, IL-1β, IL-4, IL-6, PGE2, and NO through the down-regulation of JNK/p38MAPK and NF-κB signaling, alongside reduced levels of COX2 and iNOS expression.

## Results

### BMDA and its derivative DMMA alleviate DNCB-induced AD-like phenotypes in the mice

**Figure 1:**
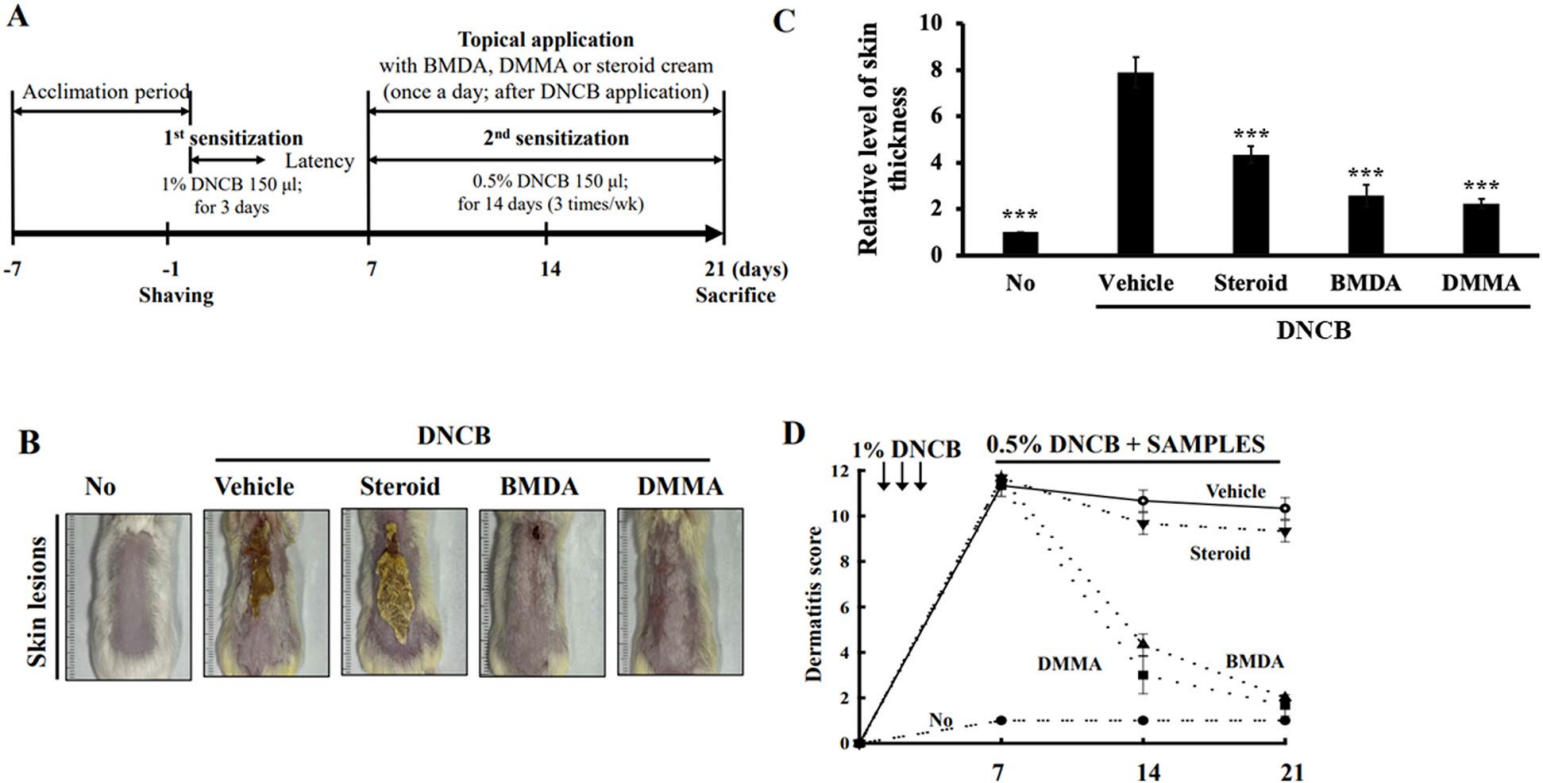
Topical application of BMDA or DMMA reduces the severity of AD-like skin lesion in DNCB-treated mice (**A**) The schedule of BMDA or DMMA treatment in DNCB-treated mice (**B**-**D**) Photographs of skin lesions from DNCB-induced Balb/C mice (n = 5 mice per group) treated with BMDA (0.3%), DMMA (0.3%), steroid (0.15%) or vehicle (a base cream) for 14 days. Representative images are shown. Skin thickness from untreated mice was measured using a caliper and served as a basis for comparison with other mouse groups. (***p < 0.001, against vehicle).

**Figure 2:**
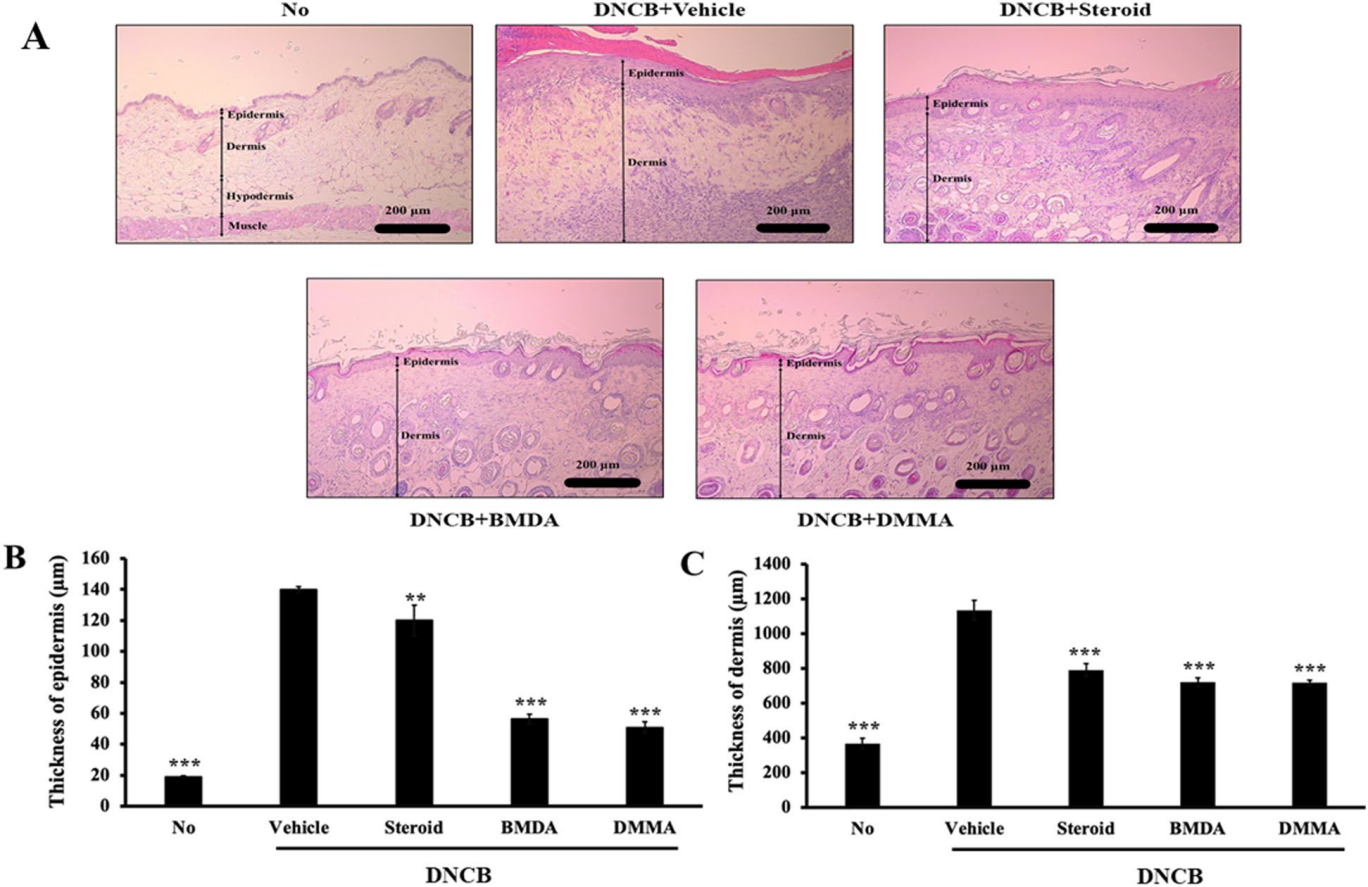
Effect of BMDA or DMMA treatment on epidermis and dermis thickness in DNCB-induced AD skin lesions (**A**-**C**) Skin lesions from DNCB-treated Balb/C mice, applied with BMDA, DMMA, steroid or vehicle for 14 days, were stained with hematoxylin–eosin solution after fixation with 10% formalin and paraffin section. The thickness of the epidermis and dermis from the mice were measured after hematoxylin–eosin staining. (**p < 0.01, ***p < 0.001, against vehicle).

BMDA and its derivative DMMA, synthesized using the reductive amination method^26^, were previously identified in our study^27^. DMMA was herein included to investigate the impact of structural variation on biological function, potentially suggesting a functional group for anti-inflammatory drug design. BMDA (0.3%) or DMMA (0.3%) was incorporated into a base cream containing corn oil, olive wax, and distilled water, as outlined in Table 1. AD-like symptoms were induced according to the scheme presented in Fig. 1A. After three consecutive treatments with 1% DNCB, 0.5% DNCB was applied to the dorsal skin three times per week for two weeks to induce AD-like symptoms. We observed that all Balb/C mice exhibited AD-like phenotypes, including hemorrhage, swelling, hives, and erosion (Fig. 1B,C). As a positive control, a steroid drug such as prednisolone valeroacetate (0.15%) was utilized. Due to the generally thinner and more sensitive nature of mouse skin compared to humans^28,29^, a steroid cream with mild potency was chosen. Notably, topical application of BMDA or DMMA on the mice's skin resulted in the recovery from hemorrhage, swelling, and erosion induced by DNCB treatment, as depicted in Fig. 1B,C. Throughout the time course for AD-like skin lesion induction, AD-like symptoms peaked seven days after the first DNCB treatment and remained consistent for two weeks (Fig. 1D). However, during BMDA or DMMA administration, AD-like phenotypes were significantly alleviated 14 days after the first DNCB treatment (Fig. 1D). Following hematoxylin–eosin staining and measurement of epidermis and dermis thickness in the mice, it was noted that BMDA or DMMA administration substantially reduced the increased thickness of epidermis and dermis caused by DNCB treatment (Fig. 2A,C). These findings indicate that BMDA and DMMA have a beneficial effect in addressing AD symptoms.

**Table 1:** Composition of cream (10 mL) for topical application on the mouse skin.

Vehicle	BMDA (or DMMA)
Distilled water 7.7 ml	Distilled water 7.7 ml
Corn oil 2.0 ml	Corn oil 2.0 ml
Olive wax 0.3 g	Olive wax 0.3 g
-	BMDA/DMMA 30 mg

### Topical application of BMDA or DMMA on the skin in DNCB-treated mice inhibits infiltration of mast cells into the skin and reduces IgE production from the sera

**Figure 3:**
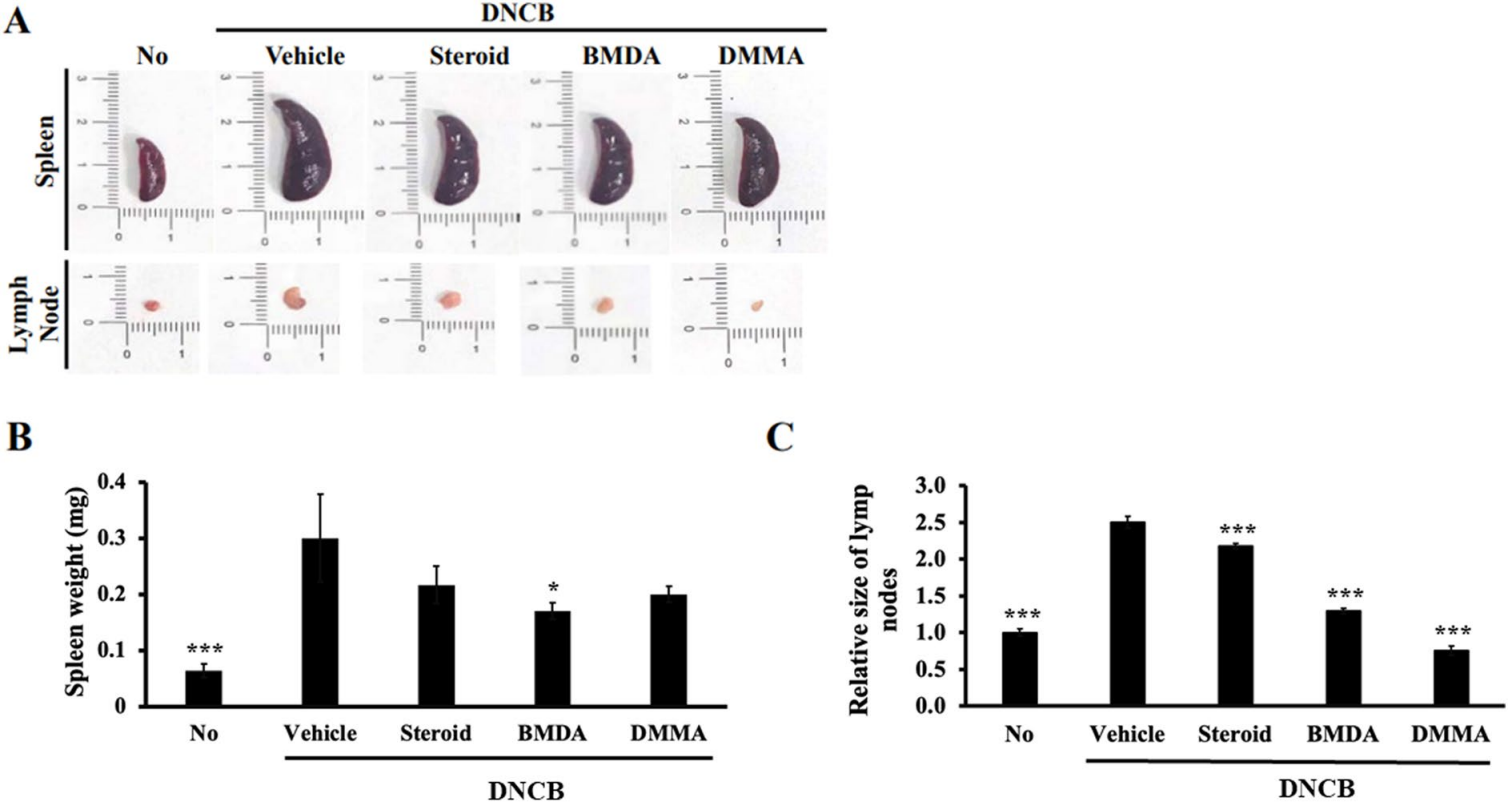
Effect of BMDA or DMMA administration on lymphoid organs in DNCB-treated mice (**A**-**C**) Spleen and inguinal lymph node sizes and weights were measured in DNCB-treated Balb/C mice, applied with BMDA, DMMA, steroid, or vehicle for 14 days. The spleen and inguinal lymph nodes were demounted for the measurements. (*p < 0.05, ***p < 0.001, against vehicle).

**Figure 4:**
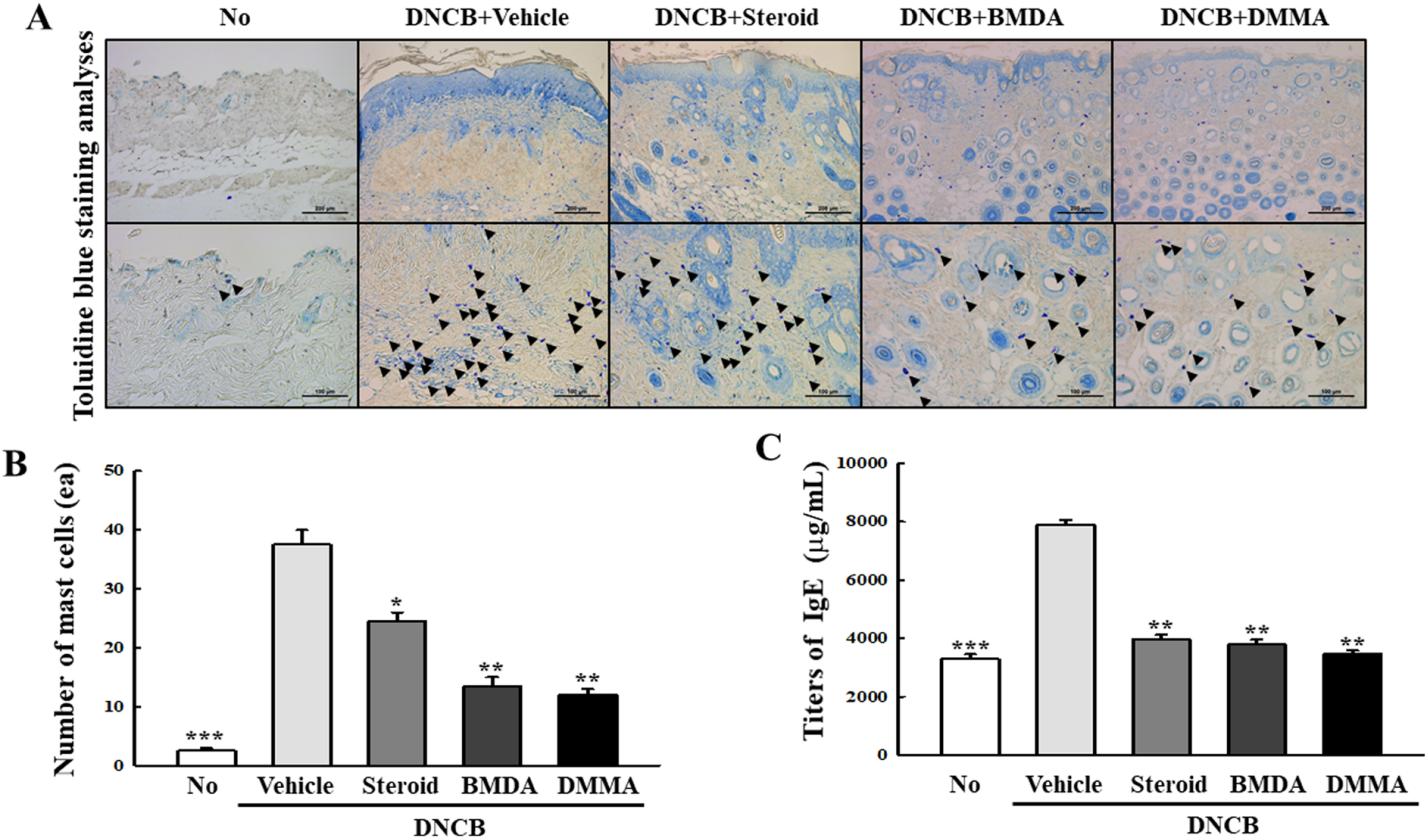
Effect of BMDA or DMMA application on allergic reaction in DNCB-treated mice (**A**–**D**) Skin lesions from DNCB-treated Balb/C mice, applied with BMDA, DMMA, steroid, or vehicle for 14 days, were stained with toluidine-blue solution after fixation with 10% formalin and paraffin section. The number of infiltrating mast cells into the epidermis and dermis was counted from five randomly selected fields under microscopy. The scale bars indicate 100 µm. Additionally, blood samples were taken from the DNCB-treated Balb/C mice, applied with BMDA, DMMA, steroid, or vehicle for 14 days, and IgE titers in the sera were thereafter measured using its specific ELISA. (***p < 0.001, against vehicle).

To explore the involvement of lymphoid organs from DNCB-treated mice in the therapeutic effect of BMDA and DMMA, we assessed the sizes of the spleen and inguinal lymph nodes in vehicle-, BMDA-, DMMA-, and steroid-treated mice during DNCB treatment. As illustrated in Fig. 3A–C, both BMDA and DMMA application significantly reduced the size and weight of the spleen, along with the size of the inguinal lymph nodes, which had expanded during DNCB treatment (Fig. 3A–C). The DNCB-induced skin disease model emulates AD, but unlike AD, DNCB induces acute inflammation. Thus, to determine whether mast cells, pivotal in the early stage of AD^6,30^, are implicated in this DNCB-induced AD-like condition, we conducted toluidine-blue staining. The results demonstrated that the infiltration of mast cells into the epidermis and dermis of DNCB-treated mice was impeded by the administration of BMDA or DMMA, mirroring the effect observed with steroid treatment (Fig. 4A,B). Additionally, given that an elevation in IgE titer is a significant immunological biomarker for the pathogenic progression of AD, we quantified IgE titers across the groups using specific ELISA. Application of BMDA or DMMA on the skin of DNCB-treated mice yielded lower IgE titers in their sera compared to vehicle application (Fig. 4C). These findings suggest that BMDA and DMMA exhibit restraint of mast cell infiltration and IgE production in DNCB-induced dermatitis.

### Administration of BMDA or DMMA decreases the expression of inflammatory cytokines, iNOS, and COX-2 transcripts in the skin of DNCB-treated mice

**Figure 5:**
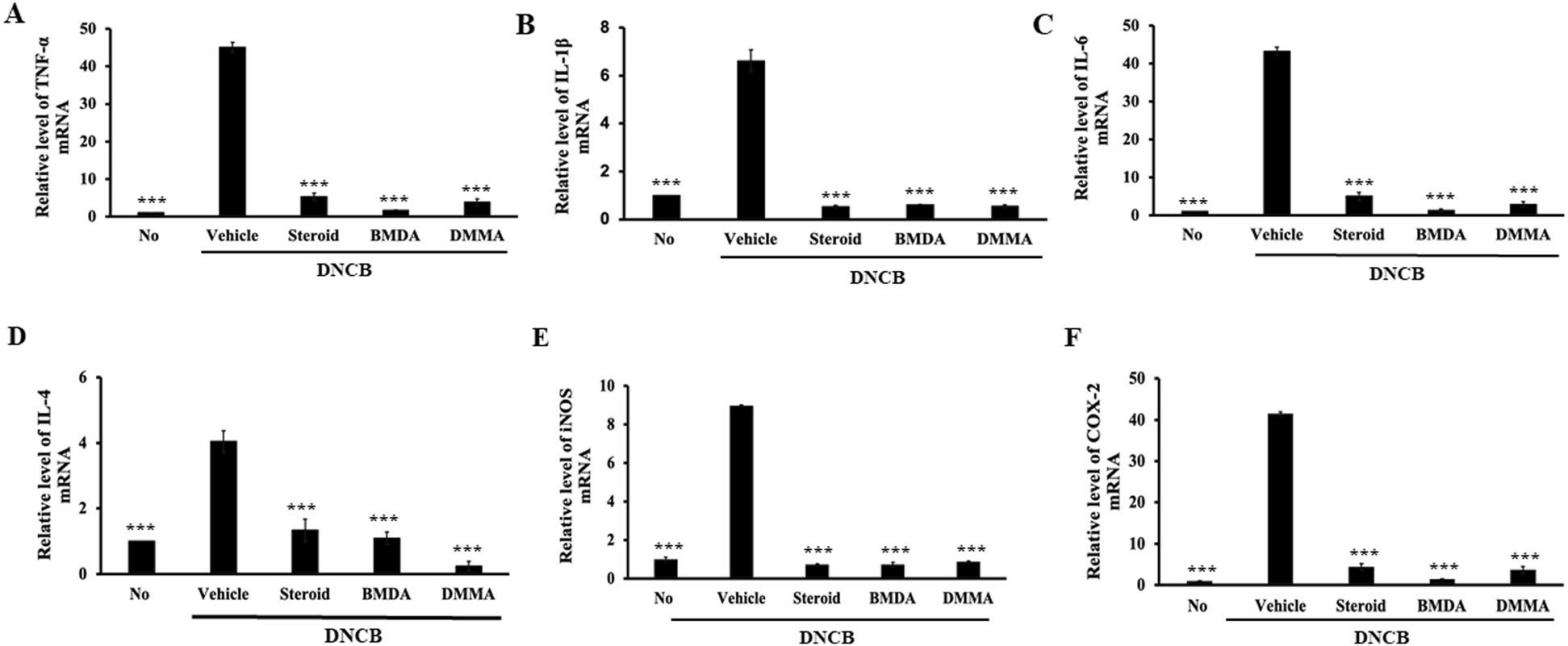
Effect of BMDA or DMMA regimen on transcript levels of inflammatory cytokines and mediator proteins in the skin from DNCB-treated mice (**A**–**F**) Transcript levels of inflammatory cytokines (TNF-1α, IL-1β and IL-6), IL-4, and mediator proteins (iNOS and COX2) were measured by qRT-PCR from the skin tissues of the mice. (***p < 0.001, against vehicle).

Given our observation of BMDA and DMMA's inhibition of immune cell activities, including mast cells and B cells (Fig. 4A–C), we directed our attention to inflammatory mediators such as cytokines, nitric oxide (NO), and prostaglandin E2 (PGE2) originating from keratinocytes and immune cells residing in skin tissues. After extracting total RNAs from the skin tissues of vehicle-, BMDA-, DMMA-, and steroid-treated mice during DNCB treatment, we quantitatively assessed the expression of inflammatory cytokines, IL-4 (type 2 immune response), iNOS, and COX2 transcripts via RT-PCR. As depicted in Fig, 5A–C, transcript levels of TNF-α, IL-1β, and IL-6 inflammatory cytokines were down-regulated by topical application of BMDA or DMMA, mirroring the effect observed with steroid treatment. In addition, the elevated expression levels of IL-4 transcript due to DNCB treatment were reduced by topical administration of BMDA or DMMA (Fig. 5D). Similarly, transcript levels of iNOS and COX2 exhibited the same trend (Fig. 5E,F). These findings suggest that BMDA and DMMA could inhibit the expression of inflammatory mediators at the transcriptional stage.

### The regimen of BMDA or DMMA diminishes the activation of MAPK and NF-κB singling pathways related to inflammation in the skin of DNCB-treated mice

**Figure 6:**
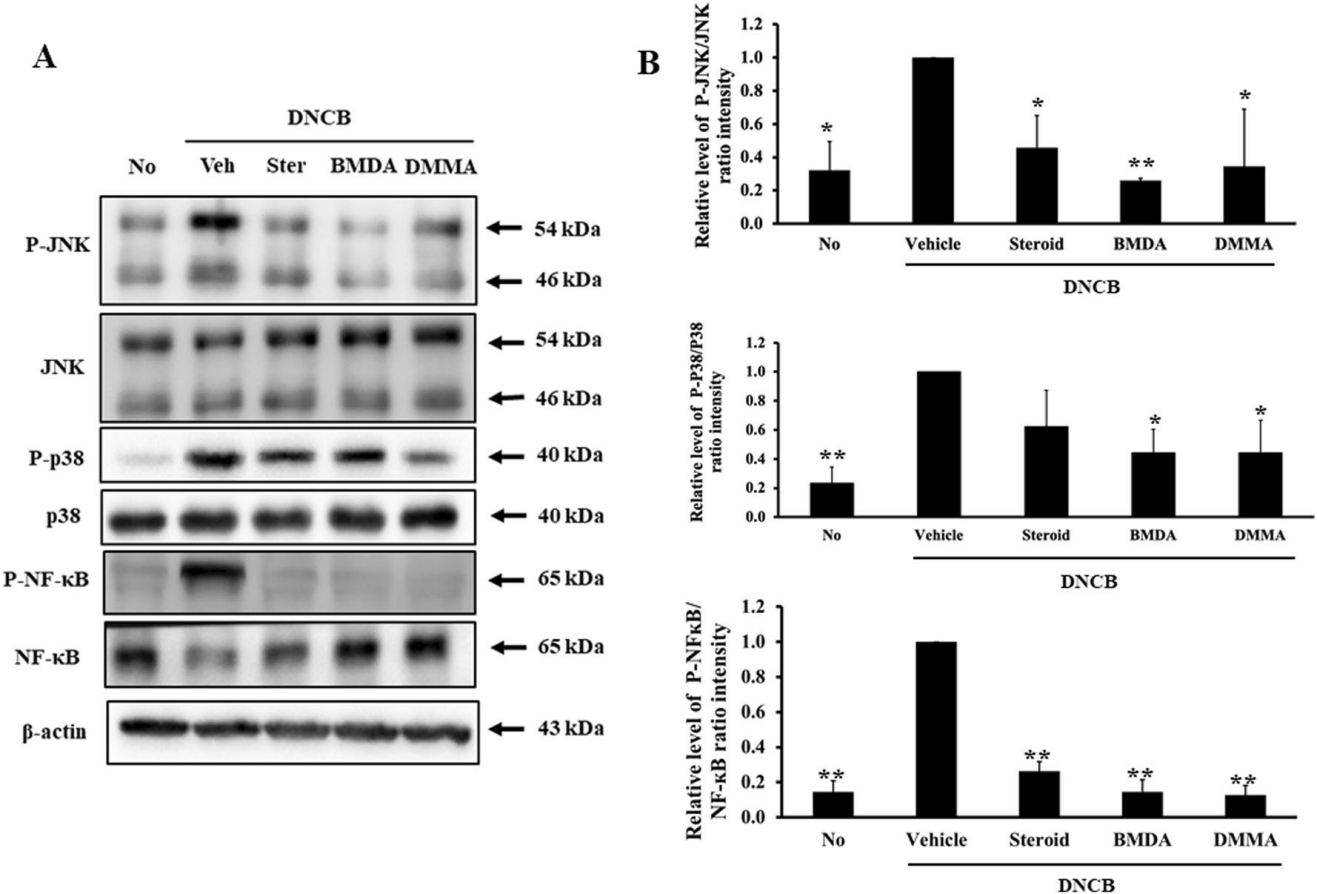
Effect of BMDA or DMMA treatment on signaling molecules involved in inflammation in the skin from DNCB-treated mice (**A**, **B**) Protein lysates from the skin tissues of DNCB-treated Balb/C mice, applied with BMDA DMMA, steroid, or vehicle for 14 days, were prepared. The phosphorylation levels of signaling molecules involved in inflammation were analyzed with immunoblotting using their specific antibodies. Phosphorylation sites of JNK indicate Thr183 and Tyr185 residues in JNK. Phosphorylation sites of p38MAPK denote Thr180 and Tyr182 residues in p38MAPK. Phosphorylation site of NF-κB (p65; RelA) designates Ser536 residue in NF-κB. The relative expression of the proteins was analyzed after scanning using AlphaView, version 3.2.2 (Cell Biosciences Inc., Santa Clara, CA, USA). (*p < 0.05, **p < 0.01 and ***p < 0.001, against vehicle).

We delved deeper into whether signaling pathways linked to the production of inflammatory mediators were influenced by the BMDA or DMMA regimen. As demonstrated in Fig. 6A,B, the application of BMDA or DMMA diminished the levels of phosphorylated JNK and p38 MAPK in the skin tissue of DNCB-treated mice. While assessing the NF-κB transcription factor, integral in the synthesis of inflammatory proteins like TNF-α, IL-1β, and iNOS, we discovered that phosphorylation levels of NF-κB were significantly inhibited through BMDA or DMMA topical application, akin to the effects observed with steroid treatment (Fig. 6A,B). These findings indicate that both BMDA and DMMA exhibit anti-inflammatory properties by deactivating JNK and p38 MAPK, along with down-regulating NF-κB.

## Discussion

**Figure 7:**
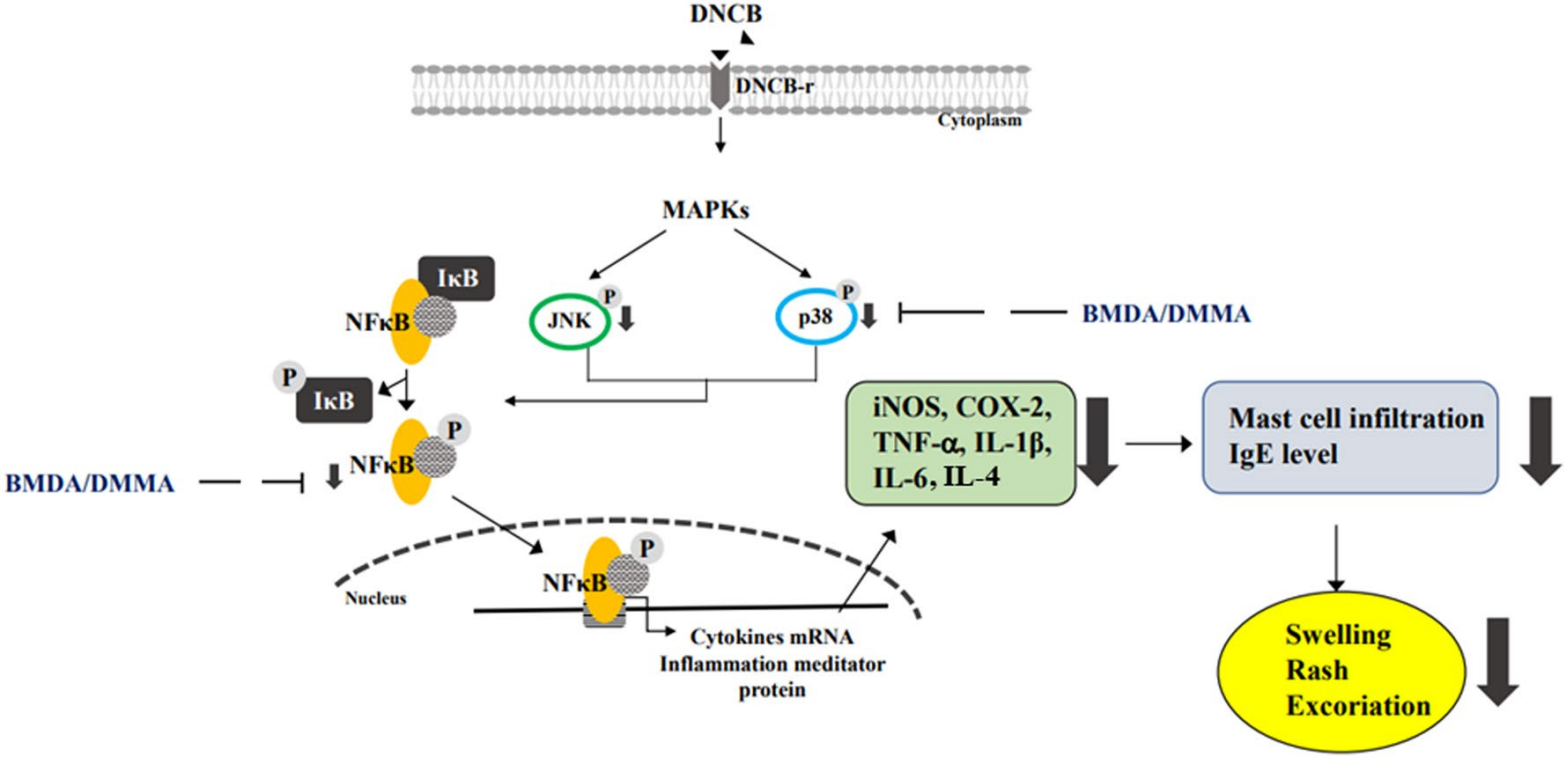
Efficacy of BMDA or DMMA on DNCB-induced inflammation in the skin of mice DNCB sensitization on the skin induces the activation of JNK, p38 MAPK, and NF-κB, resulting in an increase in the expression of inflammatory cytokines (TNF-α, IL-1β, and IL-6) and mediator proteins (iNOS and COX2). However, treatment with BMDA or DMMA inhibits the phosphorylation of JNK, p38 MAPK, and NF-κB, leading to a downregulation in the transcription of inflammatory cytokines (TNF-α, IL-1β, and IL-6), IL-4, and mediator proteins (iNOS and COX2). Consequently, this diminishes allergic reaction such as an increase of the infiltration of mast cells and IgE titer, ultimately alleviating the severity of swelling, rash, and excoriation induced by DNCB in the skin.

For the first time, we present that the topical application of BMDA and DMMA on skin tissues elicits anti-inflammatory effects on DNCB-induced AD-like symptoms. These effects can be attributed to the inactivation of p38MAPK-JNK signaling and the down-regulation of the NF-κB transcription factor. Consequently, BMDA or DMMA treatment curtailed the production of inflammatory cytokines like TNF-α, IL-1β, IL-6, IL-4 featuring a type 2 immune response, and inflammatory mediator proteins including iNOS and COX2. Based on our findings, we propose a diagram to depict anti-inflammatory effects of BMDA or DMMA on DNCB-induced AD-like symptoms (Fig. 7).

Numerous reports have extensively documented the pivotal roles of mast cells in AD^6,31,32^. Based on several lines of evidence, we speculate that repeated exposure to DNCB triggers B cells expressing IgE, resulting in IgE secretion. This IgE then binds to Fcε receptors on mast cells, prompting the release of secretory granules containing histamine and PDE2, alongside inflammatory cytokines like TNF-α, IL-1β, and IL-6. In this context, our study reveals that BMDA and DMMA effectively inhibit the infiltration of mast cells into the epidermis and dermis, as demonstrated by toluidine-blue staining. This could elucidate how these small compounds impede the inflammatory cascade reaction.

Moreover, numerous studies have documented inflammatory cytokines such as TNF-α, IL-1β and IL-6 activate JNK and p38 MAPK leading to the translocation of NF-κB to the nucleus^33,34^. In the canonical NF-κB pathway, a dimer complex composed of RelA (p65) and p50 translocates into the nucleus^35,36^. During this process, NF-κB (RelA; p65) undergoes phosphorylation at the Ser563 residue^37–39^. Given the observed upregulation of TNF-α and IL-1β expression under DNCB-induced inflammation, we assumed activation of the canonical signaling of NF-κB based on the literatures. Consequently, we noted that BMDA and DMMA reduced phosphorylation of NF-κB (RelA; p65) at Ser536 residue. These molecules also decreased the phosphorylation levels of JNK and p38MAPK. However, these results do not necessarily imply a direct targeting of JNK, p38 MAPK, and NF-κB proteins by BMDA and DMMA. Instead, we propose that BMDA and DMMA may indirectly be associated with a decrease in typical TNF-α/IL-1β-mediated inflammatory signaling. Moreover, our previous study demonstrated that BMDA and DMMA inhibited LPS-induced signaling, while enhancing NRF2 and HO-1 expression^27^. We propose that these small molecules might be involved in blocking other inflammatory signaling pathways such as TLR4-MyD88-TRAF6 signaling or NRF2-HO1 signaling. Currently, we are actively under investigation to explore target proteins for BMDA and DMMA using click chemistry techniques^40,41^.

Interestingly, upon careful comparison of the anti-inflammatory efficacy between BMDA and DMMA, DMMA displayed a slightly superior anti-inflammatory effect on DNCB-induced AD-like phenotypes than BMDA. These distinct outcomes could potentially be linked to DMMA's structural characteristics. Specifically, the inclusion of a methoxy (-OCH3) group on the benzene ring of BMDA in DMMA's chemical structure might contribute to its water solubility (hydrophilicity). Supplementary Fig. 1 illustrates that benzyl-dodecyl-methyl-amine (referred to as FMJ-G2) encompasses dodecane, differing by 2 additional CH2 groups compared to BMDA. This implies that FMJ-G2 could be more hydrophobic than BMDA, impacting its solubility and potentially its interaction with target molecules within cells. Notably, the application of FMJ-G2 did not mitigate erythema, erosion, and excoriation; furthermore, it resulted in relatively larger spleen and inguinal lymph node sizes compared to other derivatives (Supplementary Fig. 1). On the other hand, decyl-(4-fluoro-benzyl)-methyl-amine (termed as FMJ-G3), which features the attachment of a fluoride (-F) group to the benzene ring, demonstrated significant anti-inflammatory effects on DNCB-induced skin lesions. These findings strongly indicate that the observed differential anti-inflammatory efficacy may arise from variations in the chemical structures between BMDA and its derivatives. In our forthcoming study, we plan to further explore the validity of this hypothesis by conducting pharmacokinetic analysis on these derivatives.

## Materials and methods

### Antibodies and reagents

Antibodies against p38 MAPK (Cat. No. 8690 s), JNK (Cat. No. 9252 s), NF-κB (Cat No. 4764), and their phospho-specific antibodies (Cat. No. 9216 s, 9255 s, and 3033 s, respectively) were sourced from Cell Signaling Inc. (Danvers, MA, USA). For immunoblotting, anti-β-actin (Cat. No. sc-8432) antibodies were obtained from Santa Cruz Biotechnology Inc. (Santa Cruz, CA, USA). 2,4-dinitrobenzenesulfonic acid procured from Sigma-Aldrich (St. Louis, MO, USA) was used to induce AD in mice. Prednisolone valeroacetate (0.15%) was purchased from Sam- A Pharmaceutics (Wonju, Korea).

### Animal

Balb/C mice (22–25 g, 6 weeks old) were procured from Orient Bio Inc. (Seongnam, Korea). The mice were divided into five groups: untreated, BMDA, DMMA, steroid and vehicle (a base cream). Each group consisted of five animals and none died during the animal experiment. The mice were kept under specific pathogen-free conditions with unrestricted access to diet and tap water. The housing conditions included a 12-h light/dark cycle at 22 °C and 50–55% humidity.

### Synthesis of BMDA and DMMA

As previously reported^25^, BMDA and DMMA were synthesized using the reductive amination method (Molecules and Materials, Daejeon, Korea). The structures and molecular weights of the synthesized molecules were verified by H^1^-NMR, as outlined in a prior study ^27^.

### Severity scoring of atopic dermatitis and measurement of skin thickness

The severity of the DNCB-induced AD lesions was evaluated according to the SCORing Atopic Dermatitis (SCORAD) index^42,43^. Dermatitis scores 0 (no lesion), 1 (mild), 2 (moderate) to 3 (severe) were designated based on the degree of erythema, edema, excoriation, papulation, and lichenification observed on the dorsal skin of the mouse. Skin thickness was measured with a thickness gauge (Digimatic Indicator, Matusutoyo Co., Tokyo, Japan) on the last day of the experiment.

### Hematoxylin and eosin, and toluidine-blue staining

Following fixation of Balb/C mice skin tissues with 10% formalin for 48 h, the tissues were embedded in paraffin wax and sliced into 4 μm-thick slices on glass slides. The sections were stained with hematoxylin and eosin (H&E, Sigma-Aldrich) to assess skin tissue integrity or toluidine-blue solution (Sigma-Aldrich) to observe infiltrated mast cells. This was done using an optical microscope equipped with the Leica Application Suite (Leica Microsystems, Glattbrugg, Switzerland).

### Quantitative real time–polymerase chain reaction (qRT-PCR) analysis

Following the isolation of total RNAs from skin tissues, cDNAs were synthesized with Superscript II reverse transcriptase (Thermo-Fisher Scientific Inc.). As previously reported^27^, qRT-PCR was performed to measure the relative quantities of TNF-α, IL-1β, IL-4, IL-6 transcripts using 2 × Power SYBR Green (Toyobo Co., Osaka, Japan). The cytokine primer sequences were outlined in Table 2.

**Table 2:** Primer sequences used for qRT-PCR.

Inflammatory proteins	Sense primer sequence	Anti-sense primer sequence
TNF-α	5’-CCTGTAGCCCACGTC GTAGC-3’	5’-TTGACCTCAGCCTG ACTTG-3’
IL-1β	5’-GCACATCAACAAGAG CTTCAGGCAG-3’	5’-GCTGCTTGTGAGGTG CTGATGTAC-3’
IL-4	5’- TACCAGGAGCCATAT CCACGGATG -3’	5’- TGTGGTGTTCTTCGT TGCTGTGAG-3’
IL-6	5’-TTGGGACTGATGTTG TTGACA-3’	5’-TCATCGCTGTTGATA CAATCAGA-3’
iNOS	5’-TTGTGTTGGAGGTGA CCATGGAGCATC-3’	5’-CCTGTCTCAGTAGCA AAGAGGACT-3’
COX2	5’-CAGGTCATTGGTGGA GAGGTGTAT-3’	5’-CCAGGAGGATGGAGT TGTTGTAGA-3’

### Western blotting

Proteins from whole cell lysates were separated by electrophoresis on a 10–12% SDS–polyacrylamide gel and then transferred onto polyvinylidene fluoride membranes, as previously described^44^. After blocking of the blot with 5% bovine serum albumin (BSA), it was incubated with primary antibodies (1:500–1,000 dilution), followed by a horseradish peroxidase-conjugated secondary antibody (1:1,000 dilution). The membranes were visualized using an ImageQuant LAS 4000 Mini (GE Healthcare, Piscataway, NJ, USA) after the addition of a chemiluminescence solution (Pierce, Rockford, IL, USA).

### Statistical analysis

The data were presented as mean ± standard deviation (SD) and analyzed using with One-Way analysis of variance (ANOVA) followed by Tukey’s test to determine the statistical significance of the differences between groups. Error bars represent SD. A* p*-value of less than 0.05 was considered statistically significant.

### Ethical approval

Animal experiments were performed in accordance with the Laboratory Animal Resources Guide. The protocols for the animal study related to chemical-induced AD (PNU-2022–0155 for AD) were approved by the Pusan National University Animal Care and Use Committee and confirmed to the ARRIVE2.0 guidelines.

### Supplementary Information

Supplementary Information.

Supplementary Figures.

## Data Availability

The datasets used and/or analyzed during the current study are available from the corresponding author on reasonable request.

## Abbreviations

AD: Atopic dermatitis
BMDA: N-benzyl-N-methyldecan-1-amine
DMMA: [Decyl-(4-methoxy-benzyl)-methyl-amine
DNCB: 2,4-Dinitrochlorobenzene
