# Supplementary material for: N-benzyl-N-methyldecan-1-amine, derived from garlic, and its derivative alleviate 2,4-dinitrochlorobenzene-induced atopic dermatitis-like skin lesions in mice
Source: Sci Rep. 2024 Mar 21;14:6776. doi: 10.1038/s41598-024-56496-2 (PMC10958003; doi:10.1038/s41598-024-56496-2)
Supplement: Supplementary file 1 — Supplementary Information. [file 41598_2024_56496_MOESM1_ESM.docx]

**Supplementary Figure 1**

**
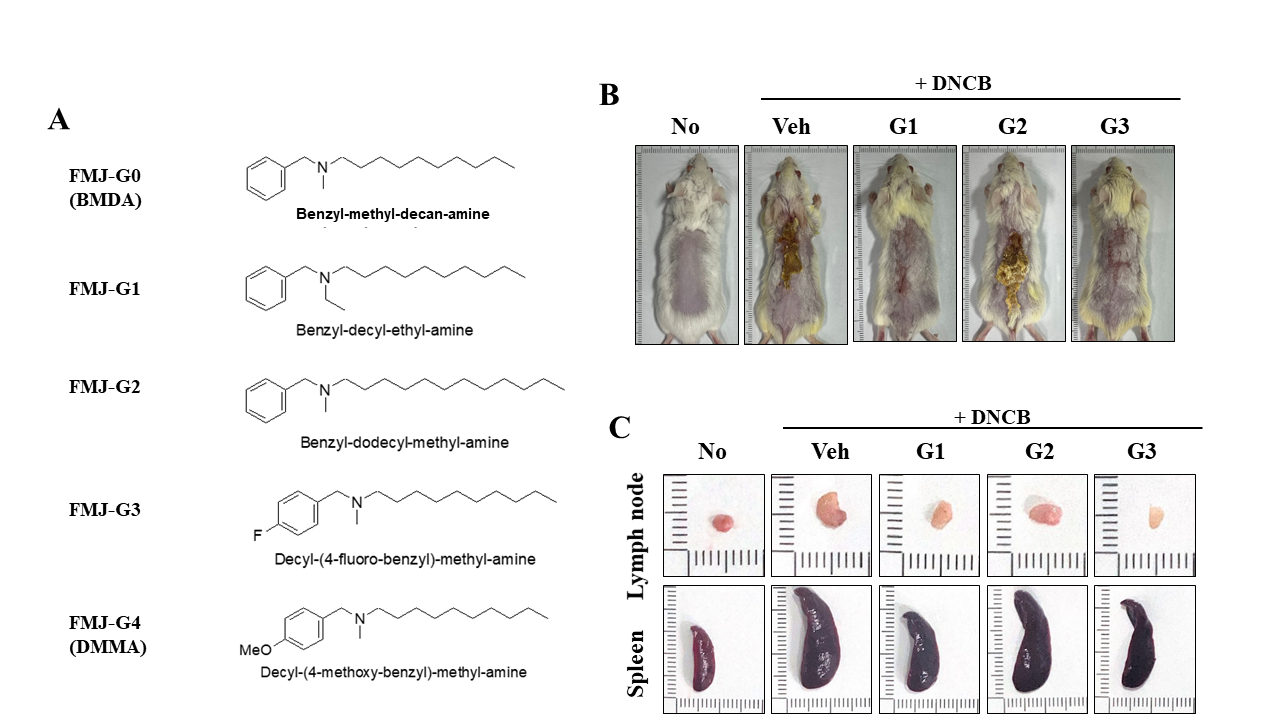
**

**Effect of BMDA derivatives on DNCB-induced AD-like skin lesion of Balb/C mice**

(A) Structures of BMDA derivatives (B) Photographs of skin lesions from DNCB-induced Balb/C mice (n=3 mice per group) treated with BMDA derivatives (FMJ-G1, -G2 or -G3) for 14 days. Representative images are shown. (C) Spleen and inguinal lymph node sizes were measured in DNCB-treated Balb/C mice, applied with BMDA for 14 days.


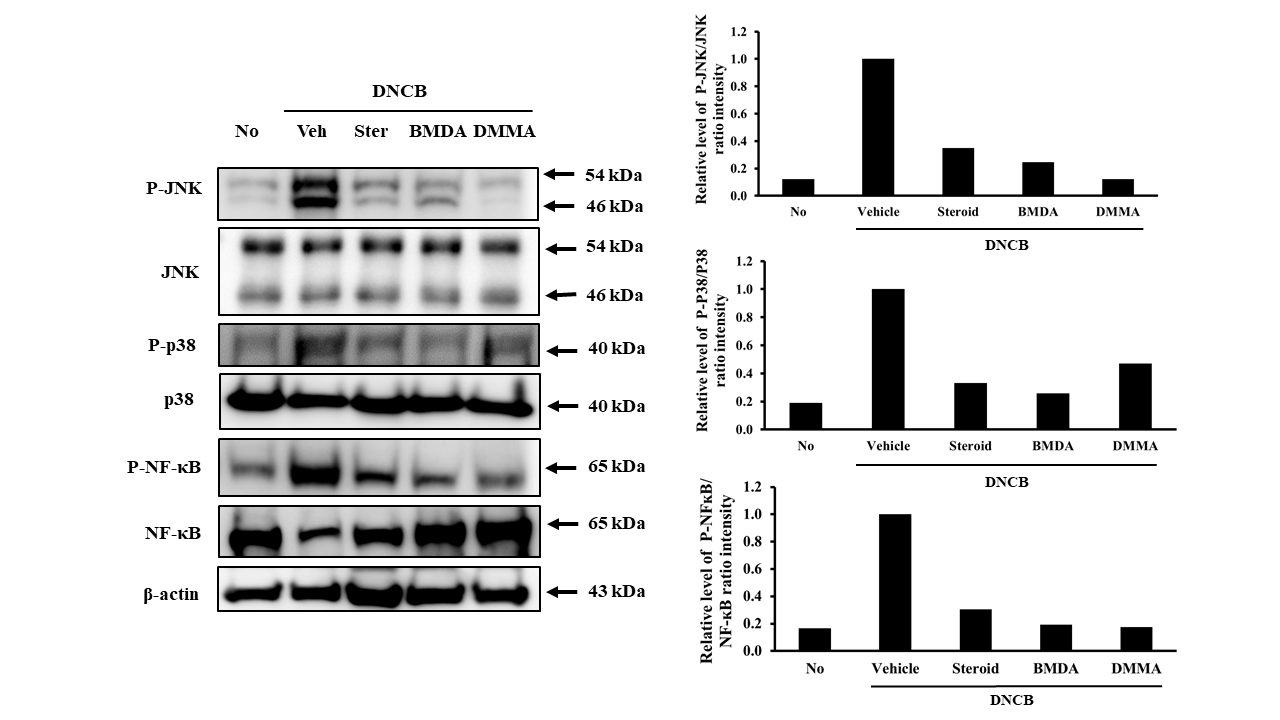
**Supplementary Figure2**


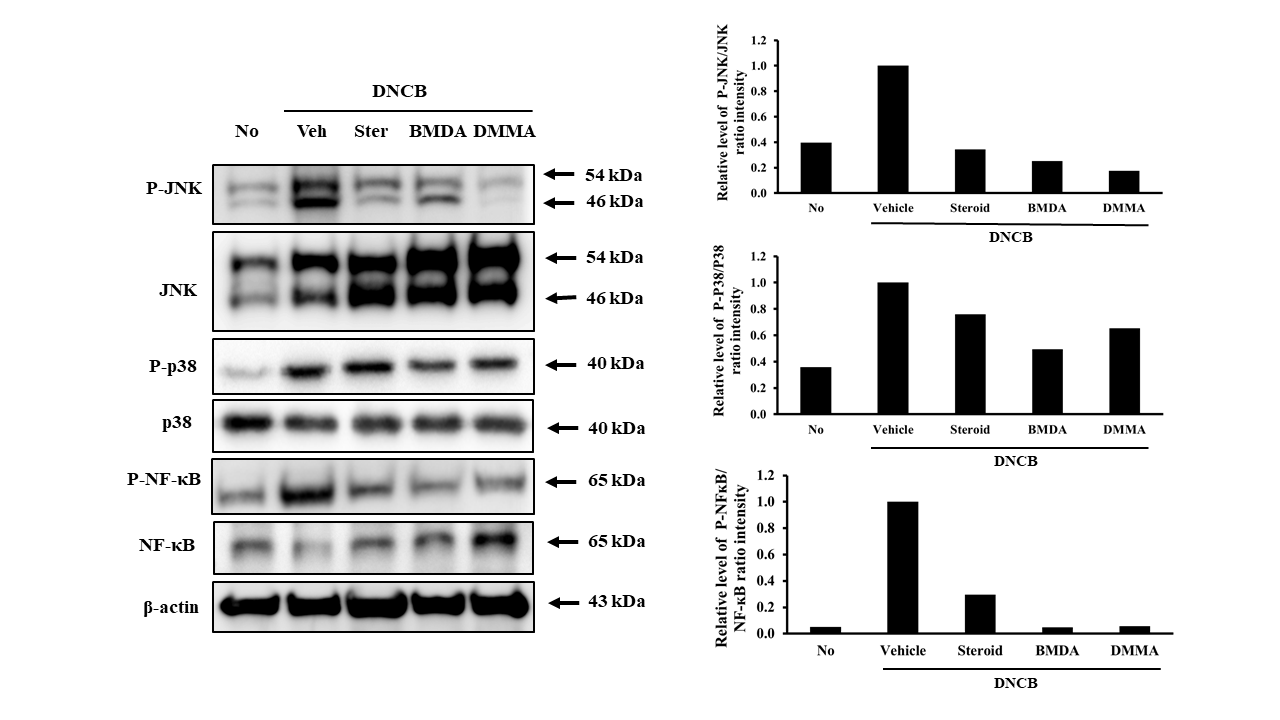


**[2^nd^ trial] [3^rd^ trial]**

**BMDA or DMMA effect on signaling molecules in DNCB-induced skin lesion**

Topical application of BMDA or DMMA decreased the phosphorylation levels of signaling molecules (JNK, p38MAPK, and NF-κB) involved in DNCB-induced skin lesion. The phosphorylation levels of signaling proteins (JNK, p38MAPK, and NF-κB) were analyzed with immunoblotting using their specific antibodies. The immunoblotting blotting was repeated.
