# Supplementary figures and images for: N-benzyl-N-methyldecan-1-amine, derived from garlic, and its derivative alleviate 2,4-dinitrochlorobenzene-induced atopic dermatitis-like skin lesions in mice
Source: Sci Rep. 2024 Mar 21;14:6776. doi: 10.1038/s41598-024-56496-2 (PMC10958003; doi:10.1038/s41598-024-56496-2)

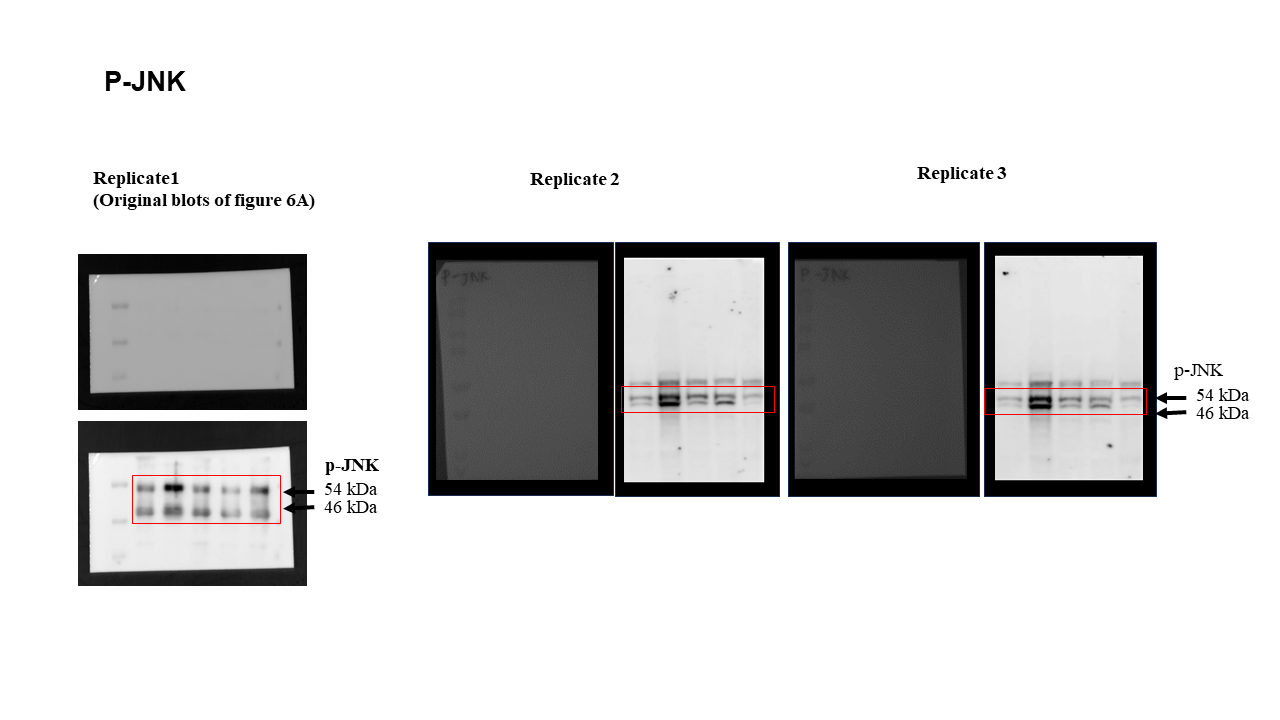


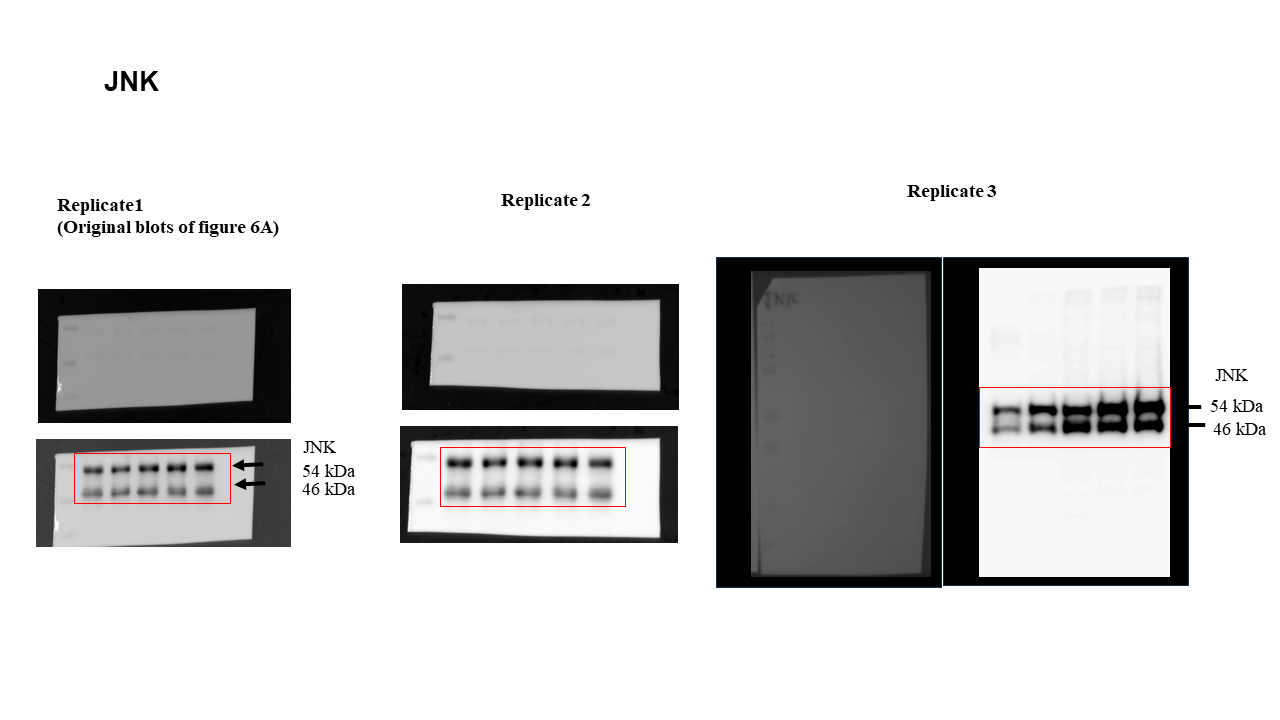


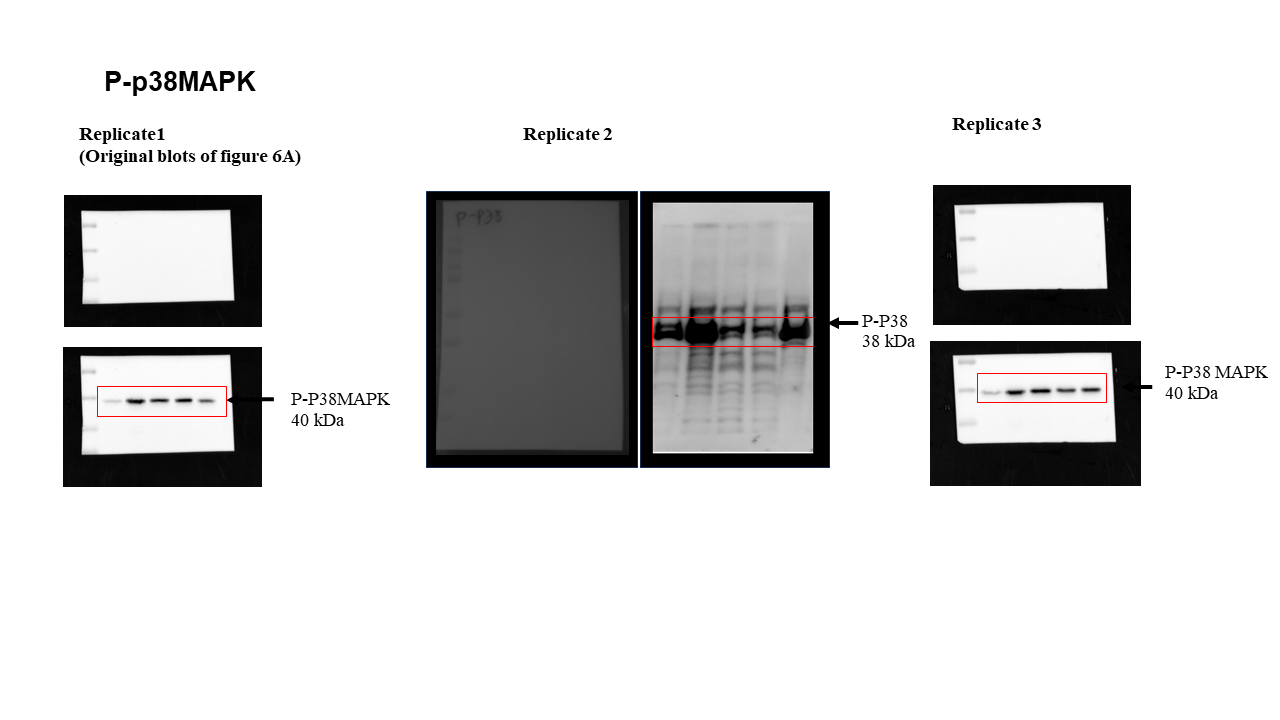


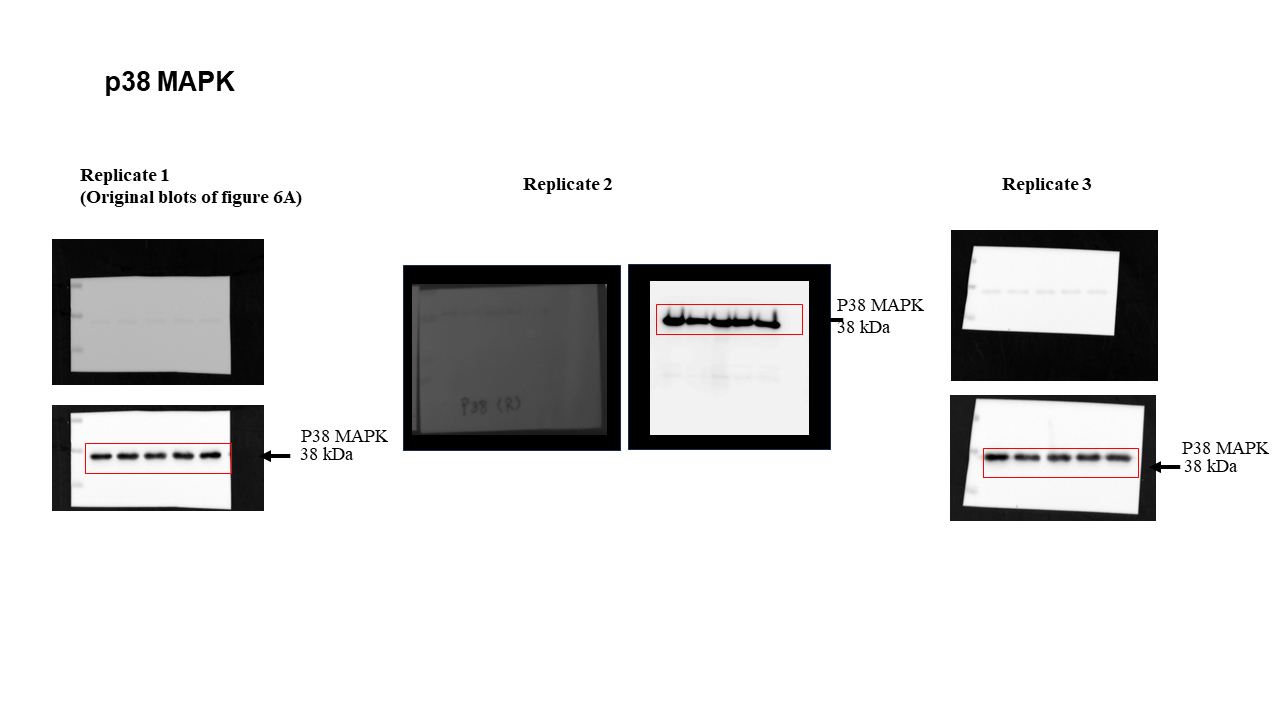


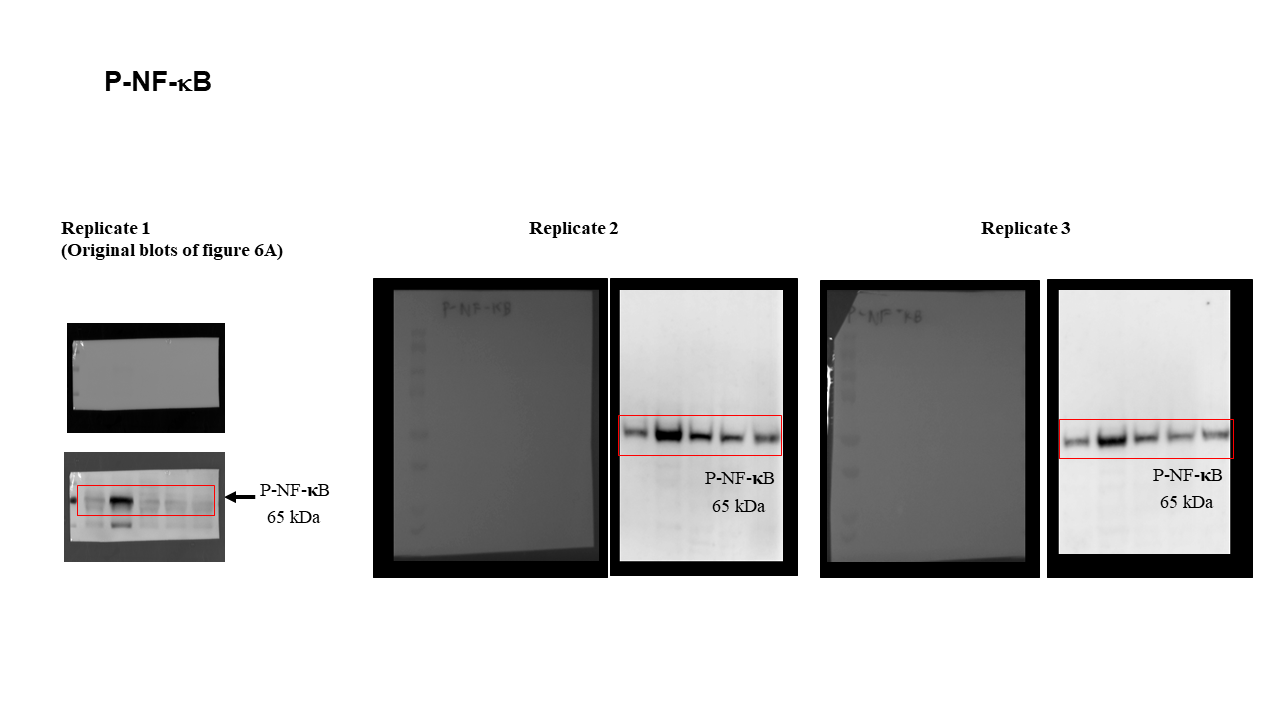


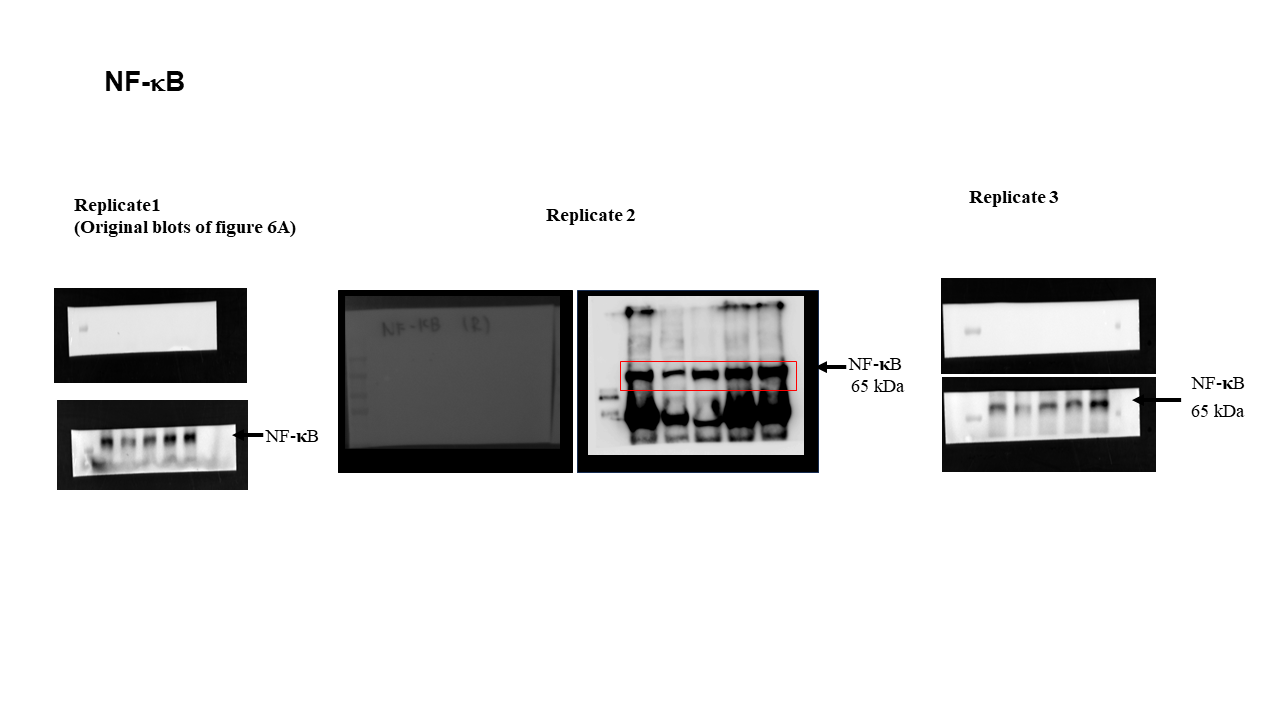


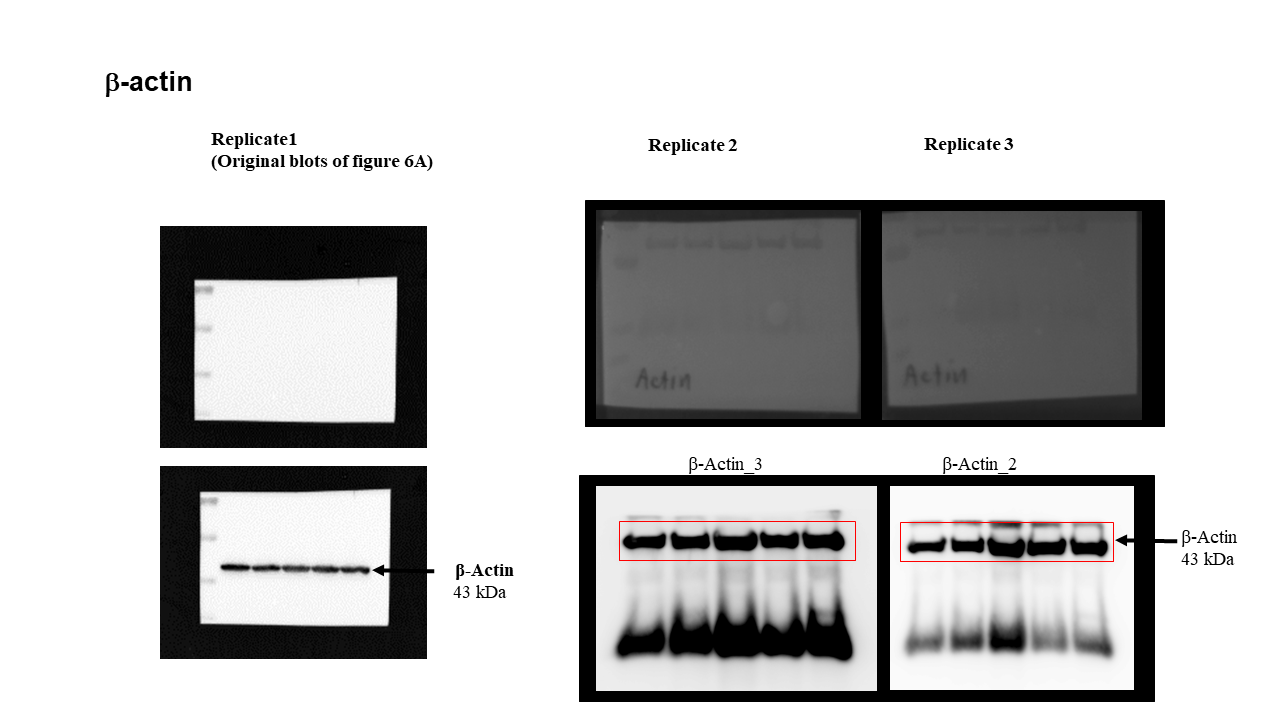

Supplement: Supplementary file 2 — Supplementary Figures. [file 41598_2024_56496_MOESM2_ESM.docx]
